# Long-Term Follow-Up After Non-Curative Endoscopic Submucosal Dissection for Early Gastrointestinal Cancer—A Retrospective Multicenter Analysis

**DOI:** 10.3390/jcm13216594

**Published:** 2024-11-02

**Authors:** Philipp Pimingstorfer, Matus Gregus, Alexander Ziachehabi, Reinhold Függer, Alexander R. Moschen, Rainer Schöfl

**Affiliations:** 1 University Clinic for Internal Medicine 2, Kepler University Clinic, 4020 Linz, Austria; 2 Medical Faculty, Johannes Kepler University, 4020 Linz, Austria; 3 Department for Internal Medicine 4, Ordensklinikum Linz—Barmherzige Schwestern, 4020 Linz, Austria; 4 Department for Surgery, Ordensklinikum Linz—Barmherzige Schwestern, 4020 Linz, Austria

**Keywords:** non-curative ESD, ESD, non-curative

## Abstract

**Background**: Endoscopic Submucosal Dissection (ESD) has become the standard therapy for early malignant lesions in the gastrointestinal tract and has shown as good oncological surgery results. Approximately 30% of ESDs do not meet the criteria for oncological curability, and upfront surgery is indicated. Hence, about 40% of patients with an indication for surgery are advised against surgery because of comorbidities and an advanced age. **Methods**: We performed a multicenter retrospective cohort study on the long-term outcomes of non-curative ESDs, performed between 2009 and May 2024, without additional tumor therapy. The primary outcome was the recurrence of malignancy, either local malignancy or lymph node metastasis during follow-up, or death. We compared the outcomes between two cohorts: after non-curative ESD (ncESD) and after curative ESD (cESD). **Results**: A total of 374 ESDs were analyzed in this study. Overall, the technical success rate was 91%, and the oncological curative resection criteria were met in 70.9% of patients. Severe complications occurred in 5% of cases without procedure-associated mortality. In the ncESD group, 20% (7/35) of patients had a recurrence of malignancy primarily due to positive horizontal margins in the resection specimens, and 3 out of 35 died due to a non-oncological reason during the follow-up (mean length 36.6 months). In the cESD group, 3% (1/33) of patients had tumor recurrence, and 1 patient died because of a non-oncological reason. The tumor recurrence rate between the cohorts was significant (*p* = 0.017), and overall mortality did not show significance (*p* = 0.33). **Conclusions**: Especially in the elderly and multimorbid patients, the recommendation to perform rescue surgery after non-curative ESD remains challenging. Residual malignancy rates in surgical resection specimens are low, recurrence rates of malignancy are low, and mortality rates for non-oncological reasons are high in this population. There is a need for more data for the individualization of patient management after non-curative ESD.

## 1. Introduction

With evolving therapeutic strategies, endoscopic resection has become the standard therapy for early esophageal, gastric, and rectal malignant lesions not only in Asia, but also in Western countries, including most of Europe. The technique of Endoscopic Submucosal Dissection (ESD) has shown to have oncological results as good as surgery in combination with a favorable safety profile in early gastrointestinal cancer [1,2,3,4,5].

Whether ESD is a curative intervention or not depends on several histopathological risk factors in the resection specimen, such as the R-stage, tumor differentiation, invasion depth, the infiltration of vessels, or lymphovascular infiltration [6,7,8,9,10,11,12,13,14]. These criteria are associated with the risk of synchronous lymph node metastasis or lymphatic cancer recurrence. The determination of curative ESD follows the main principle that the risk of recurrence by lymph node metastases must be lower than the risk of death following major surgery.

Surgery after non-curative ESD is as safe as first-line surgery without a higher probability of major complications [15,16]. However, due to the advanced age and multimorbidity of some patients, interdisciplinary tumor conferences recommend refraining from surgery in up to 40% of cases [15]. As early gastrointestinal cancer often concerns elderly patients and demographic changes in European and East Asian populations, this scenario will become even more common in the future.

As data on the long-term outcomes of non-curative ESD are scarce, we aimed to evaluate the recurrence of malignancy or death (tumor-related and non-tumor-related) after non-curative ESD in early esophageal, gastric, and rectal cancers. Therefore, we performed a retrospective multicenter cohort analysis in the federal state of Upper Austria. To our knowledge, no Austrian data on this topic have been published so far.

## 2. Methods

We performed a multicenter retrospective cohort analysis of all ESDs performed in esophageal, gastric, and rectal lesions between 2009 and May 2024. Participating hospitals included Ordensklinikum Linz—Barmherzige Schwestern; Ordensklinikum Linz—Elisabethinen; and the Kepler University Clinic.

To identify all ESDs, we searched each hospital’s database for ESD billing data provided to social insurance. After the exclusion of primary intended piecemeal snare resections and billing errors, a combined database of all ESDs was created using Microsoft Excel 2016. For data protection, we used pseudonymization in the database. We noted the ages and sexes of patients. To assess the fitness of patients, we used the American Society of Anesthesiologists (ASA) Score [17].

For clinical classification of lesions, we noted lesion size and localization. Technical success of ESD was defined as en bloc resection without visual residual malignancy and without perforation. Complications were noted in the database. Severe bleeding was defined as overt clinical signs of bleeding (melena or hematemesis) that required subsequent therapeutic endoscopy. Perforation was defined as a visible lesion during the ESD procedure or clinical symptoms in combination with free air in a computed tomography scan after ESD. Intramural air without symptoms was not counted as a perforation.

We checked the histopathologic results and distinguished between malignant and non-malignant lesions. We defined adenomas with low- or high-grade dysplasia as well as esophageal lesions with low- or high-grade dysplasia as non-malignant. For the purpose of this study on mucosal cancer, we excluded ESD for atypical indications such as Neuroendocrine Tumors (NETs) or Gastrointestinal Stroma Tumors (GISTs).

For malignant lesions, we defined curative resection or non-curative resection using established criteria from the ESGE guidelines [18]. The criteria for the curative resection of early gastrointestinal cancer comprise histopathological characteristics and technical standards.

First, technical success and, therefore, the criterion for curative resection is defined as en bloc resection of the lesion with malignancy-negative horizontal and vertical margins of the resection specimen without periinterventional perforation.

Second, the risk of lymph node metastasis differs between organs, and an oncological curative resection is defined for the following:

- Squamous cell carcinoma in the esophagus: pT1b (sm1) (as expanded indication) with an invasion depth < 200 µm in the submucosal layer without risk factors (L0 and V0) and a tumor differentiation of G1 or G2.
- Barrett´s carcinoma or carcinomas of the esophagogastric junction: pT1b (sm1) with an infiltration depth of up to 500 µm in the submucosal layer, L0, V0, and a tumor differentiation of G1 or G2.
- Gastric carcinoma: A differentiated non-ulcerated tumor < 2 cm diameter and L0 and V0 with a maximum of one of the expanded criteria, namely pT1b (sm1) with an infiltration depth of up to 500 µm in the submucosal layer, a non-ulcerated lesion independent of size, a differentiated ulcerated lesion < 3 cm diameter, or an undifferentiated lesion < 2 cm in diameter.
- Rectal cancer: Adenocarcinomas pT1b (sm1) with an infiltration depth of up to 1000 µm in the submucosal layer, L0, V0, a tumor differentiation of G1 or G2, and tumor budding ≤ 1.

Regarding patients with malignant histology, we discussed a patient in the interdisciplinary tumor conference independent of curative or non-curative ESD. In non-curative cases, we noted the recommendation for further therapy or follow-up due to advanced age or multimorbidity.

In patients with positive vertical (= lateral) margins in the resection specimen as the only risk factor, the guidelines recommend a close follow-up [18] because data show that only a small proportion of patients have residual cancer [19]. These patients were excluded from this analysis (n = 13).

**Figure 1:**
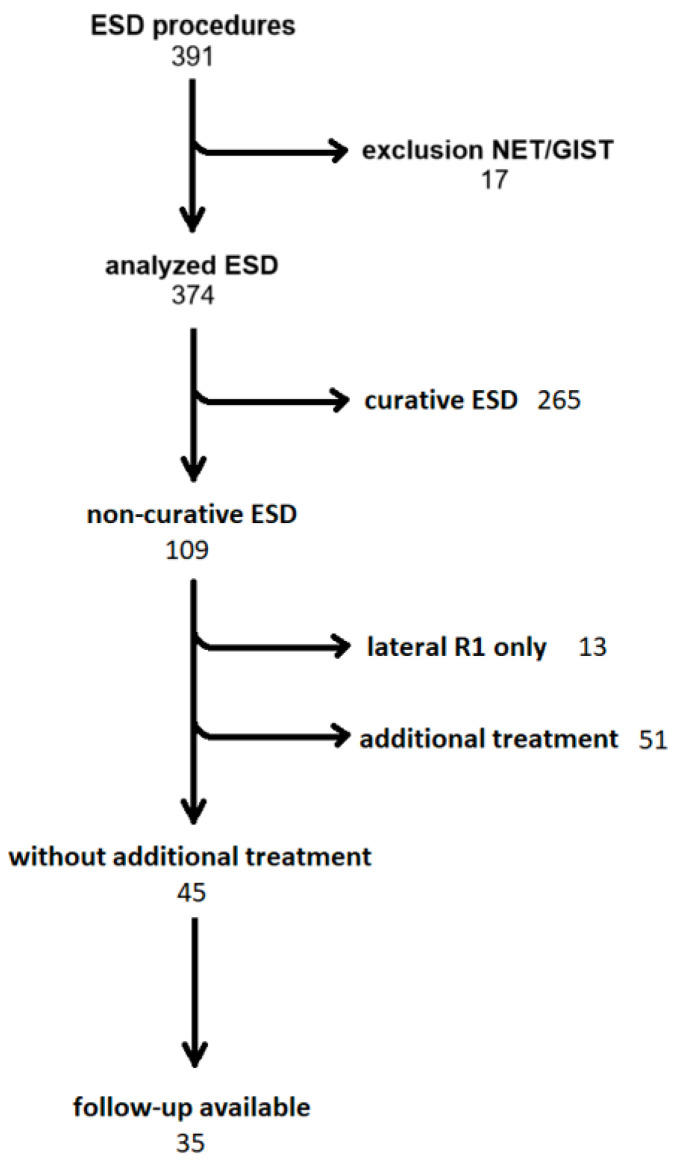
Recruitment of patients for data analysis. Abbreviations: ESD—endoscopic submucosal dissection; NET—neuroendocrine tumor; GIST—gastrointestinal stroma tumor.

Follow-up data in available cases were noted in the database. Figure 1 shows an overview of the process of data collection.

Before ESD, each patient had a second-look endoscopy by an expert endoscopist to check the indication and feasibility of ESD using optical criteria. In potentially advanced lesions, a staging endoscopic ultrasound was performed. If the lesion was classified as non-eligible for ESD (e.g., ulceration, signs of deep submucosal infiltration via vascular pattern, involvement of muscular layer, or positive lymph nodes in endoscopic ultrasound), ESD was not performed. All patients provided informed consent for the ESD procedure. ESDs were primarily performed by one expert endoscopist in combination with two trainees, with one in each center.

Since this is a retrospective real-world analysis, follow-up examinations were individualized for each patient according to histopathology and local expertise. Generally, patients with curative ESD had follow-up endoscopies every three months in the first year, every six months in the second and third year, and yearly thereafter. In patients with non-curative ESD, complimentary endosonography was added at each follow-up visit. We did not perform computed tomography (CT) or positron emission tomography (PET) routinely in all patients.

We performed optical diagnosis in combination with targeted biopsies to capture local recurrence. We defined local recurrence as histologic proof of high-grade dysplasia or carcinoma. In such cases, especially in Barrett´s esophagus, we distinguished between true local recurrence and metachronous lesions in other areas of Barrett´s esophagus. In suspected lymph node recurrence, we added a computed tomography scan and discussed additional diagnostic or therapeutic measures in the interdisciplinary tumor conference.

As all participating hospitals are tertiary care centers, a certain proportion of patients were referred for ESD. Regarding these referred patients, rescue surgery as well as follow-up was performed in the referring hospital. We did not request outcome data for patients who had a follow-up after ESD in other hospitals.

To compare the long-term outcomes of patients after undergoing non-curative ESD with those of patients after undergoing curative ESD, we created the following two cohorts of patients, and each had available follow-up data: the non-curative ESD (ncESD) group without additional therapy, like surgery or radio-chemotherapy, and the curative ESD (cESD) group which initially had a malignant histopathologic result.

This study was approved by the ethics committee of Johannes Kepler University Linz (1224/2024).

### Statistical Methods

For database creation and descriptive statistical analysis, Microsoft Office Excel 2016 was used. In descriptive statistics, continuous variables are stated as the mean and standard deviation of the mean (SD). Advanced statistical analysis was performed using IBM SPSS Statistics 29.0.2.0. All data of continuous variables were checked for normal distribution (Kolmogorov–Smirnov with Lilliefors significance correction, type I error = 10%). As none of the continuous variables showed normally distributed data, the exact Mann–Whitney U test was used for cohort comparisons. Dichotomous variables were compared using Fisher’s exact test and the chi-square test. Statistical significance was defined by a *p*-value ≤ 0.05.

## 3. Results

A total of 391 ESDs were identified in participating hospitals during the observation period. After the exclusion of 17 ESDs for atypical indications like NET and GIST, we included 374 ESDs for mucosal lesions in our analysis.

The ESD procedures comprised 131 esophageal ESDs, 103 gastric ESDs, and 140 rectal ESDs. In esophageal lesions, 81% (n = 106) of ESDs were performed for Barrett-associated lesions, and 19% (n = 25) were for squamous epithelial lesions. The mean age of the patients was 71.3 years (SD 12.6), and 72% were male. The mean specimen size was 40.1 mm (SD 20.9), and the mean duration of ESD was 138 min (SD 105). The technical success rate was 91%. Regarding the oncological outcome, 70.9% of ESDs showed curative histopathologic results (n = 265). In esophageal lesions, the curative resection rate was 56%; in gastric lesions, it was 70%; and in rectal lesions, it was 81%.

Severe complications, defined as perforation or bleeding that required subsequent endoscopy, occurred in 5% of cases (n = 18), which included 11 bleedings and 7 perforations. The bleedings were all successfully managed via endoscopy.

A total of 29.1% of ESDs were non-curative (n = 109). After discussion in the interdisciplinary tumor conference, 45 of those patients (41.3%) were advised against additional therapy, like surgery or radio-chemotherapy, due to comorbidities. Surgery was performed in 42 cases, and radio-chemotherapy was performed in 9 cases. Thirteen non-curative ESD cases with residual malignancy in vertical resection specimen margins (vertical R1) as the only risk factor were excluded from further analysis.

The rate of residual malignancy in resection specimens of oncologic surgery after non-curative ESD was 29% (12/42). Severe surgical complications (Dindo-Clavien ≥ 3) occurred in 22% of esophageal surgeries and in none of the gastric or rectal surgeries. Perioperative 30-day mortality did not occur.

Follow-up data of the 45 patients with non-curative histopathological results without additional therapy were available in 35 cases. These patients comprised the ncESD group.

The mean age was 77 years (SD 11.3), and 80% were male. The mean ASA Score of the patients was 2.4. ESDs were performed for esophageal carcinomas in 16 cases (three squamous cell carcinomas), for gastric lesions in 10 cases, and nine ESDs were performed in the rectum. Table 1 provides an overview of the histopathologic results of those patients and the reasons for non-curative ESD. The main reason for non-curative resection was deep submucosal infiltration (>sm1) and positive horizontal margins in resection specimens throughout the organs. In esophageal lesions, 50% of non-curative lesions showed a poorly differentiated tumor (G3).

The mean follow-up period was 36.6 months (SD 27.1). Malignant recurrence, defined as biopsy-proven high-grade dysplasia or carcinoma, was observed in seven cases, including six local recurrences and one patient that had lymph node metastasis.

All six patients with local recurrences underwent subsequent endoscopic therapy. Three patients died during the observational period for non-oncologic reasons (one due to pneumonia; two due to major cardiovascular events). Table 2 shows the histopathology of the resection specimens of patients with tumor recurrence. Notably, most recurrences occurred after positive horizontal margins in the initial resection specimen, and lymphatic recurrence occurred due to an undifferentiated tumor type.

Of the 265 patients with curative ESD, follow-up data in patients with malignant histopathology were available in 33 cases. Most patients after curative ESD had a follow-up in the referral hospital from which we did not request data. These patients comprised the cESD group. Table 3 shows the baseline characteristics and outcomes of the ncESD and cESD cohorts.

The mean age of patients in the cESD cohort was 72.9 years (SD 11.1), and 73% were male. The mean ASA Score of patients was 2.2. ESD was performed for early esophageal carcinomas in 18 cases (two squamous cell carcinomas), 11 ESDs were performed for early gastric cancer, and 4 were performed for early rectal cancer. The mean observational period was 16.2 months (SD 12.1).

Local malignant recurrence occurred in one patient; no cases of lymph node metastasis were observed during the observational period. In two patients, metachronous lesions in Barrett´s esophagus were detected in other locations than the initial ESD scar despite subsequent radiofrequency ablation therapy being carried out. All three lesions were managed successfully via ESD. One patient in the cESD cohort died during the observational period because of a major cardiovascular event.

In the comparison of the cohorts, there was a trend towards patients having a higher age in the ncESD group without additional therapy (*p* = 0.12). The ASA Score showed no significant difference between the cohorts. The mean available follow-up period showed a difference between the cohorts due to the methodology.

There was a significant difference between the cohorts regarding tumor recurrence, which had a lower rate in the cESD group (*p* = 0.017). No malignancy-related deaths were observed in the observational periods in both groups. There is a trend towards higher mortality in the ncESD group (*p* = 0.33) because of comorbidities, although the group size did not show significance in this retrospective analysis. No difference regarding tumor-associated mortality was found between the cohorts.

## 4. Discussion

Especially in elderly and multimorbid patients, the recommendation to perform surgery or additional therapy, like radio-chemotherapy, remains challenging, although surgery after ESD seems to be safe regarding major complications [16].

### 4.1. Limitations

For our study, we must report a selection bias for the cESD cohort because follow-up data on curative ESD were only available in ESDs performed between 2020 and the end of the observation period. Therefore, the follow-up interval is significantly shorter than that in the ncESD group. This fact must be considered when interpreting the comparative results between the cohorts. Furthermore, the sample size is too small to account for a meaningful subgroup analysis.

As our study on the long-term outcomes after non-curative ESD without additional therapy is a retrospective analysis, it shows real-world practices of follow-ups and outcomes in non-curative ESD in a multimorbid patient cohort. Due to the lack of a standardized follow-up study protocol, there is a risk of underestimation of lymph node recurrence. But in clinical practice outside of clinical trials, there is a conflict regarding the modalities of follow-up examinations because patients that did not undergo additional surgery after non-curative ESD were not eligible for surgery due to a comorbidity in the first place. Hence, follow-up examinations were performed without a computed tomography scan or endosonography in some cases because of lacking therapeutic consequences. To avoid this follow-up bias, large prospective controlled trials are necessary to provide information on which patients can be advised safely against additional surgery. Furthermore, data on mortality rates in both cohorts are scarce in our study because we did not contact patients without eligible data in participating hospital databases as this is a retrospective non-interventional study.

### 4.2. Comparison to Published Data

The overall outcomes of ESDs in Upper Austrian centers are in line with the international published literature [20,21]. The rate of residual malignancy in surgical resection specimens after non-curative ESD in Upper Austrian centers is low [15], which is in line with the published literature [22].

Our data are in line with the published literature showing that the recurrence of malignancy occurs only in a small proportion of cases. Santos-Antunes et al. [22] showed that in a similar study of 33 patients that underwent non-curative ESD without additional surgery, the recurrence rate was 13% in a cohort of patients with early esophageal, gastric, duodenal, and rectal cancers.

A study [23] showed that in rectal lesions, residual malignancy occurs in 19% of cases after non-curative ESD in surgical resection specimens. During the follow-up, the disease-specific survival rates did not differ between the surgical group and the follow-up group. Only 20% of patients with esophageal adenocarcinomas after non-curative ESD had tumor recurrence during the follow-up according to a small study in the United States [24]. A recent study on early gastric cancer [25] showed similar recurrence rates after non-curative endoscopic resection.

All perforations in our cohort occurred in gastric lesions in the first five years of the observation period, which may represent the learning curve in this technique. The ASA Score did not show a difference between the cohorts even though patients in the ncESD cohort were not fit for surgery. This shows that patients in the cESD group were comorbid as well, and comorbidity per se does not count as a criterion of curative resection.

Our data show that malignancy-related morbidity in ncESD is low, and ESD can prevent cancer-associated death, even if patients do not met the criteria of curativeness. Further data on this topic are necessary.

### 4.3. Individualized Multidisciplinary Approaches

These results emphasize the need for individualized decision making on whether to perform surgery or not. Several histopathologic studies show that tumor differentiation and lymphovascular infiltration are independent risk factors for lymph node metastasis [6,26]. In these patients, the risk of malignancy recurrence exceeds the risk of surgical complications, and surgery should be performed in eligible patients.

In contrast, an isolated tumor infiltration depth of >sm1 without lymphovascular infiltration does not seem to be an independent risk factor for lymph node metastasis [27]. In colorectal lesions, the risk of lymph node metastasis seems to be around 6%, which must be considered in decision making, and it is lower than the perioperative risk for complications but not for death. Due to the higher density of lymphatic vessels in esophageal and gastric mucosa and submucosa, the infiltration depth of early cancer seems to be an independent risk factor for lymph node metastasis in these lesions. The risk of tumor recurrence in gastric lesions seems to be higher than the risk of surgical complications; therefore, surgery is recommended [26].

Because of manifold data on risk assessment for lymph node metastasis and the surgical risk, Hatta et al. [28] developed a scoring system (eCura) to stratify curability after non-curative ESD, defined by current guideline criteria. Five risk factors are weighed with point values and are then categorized into three groups: low risk (0–1), intermediate risk (2–4), and high risk (5–7). Cancer-specific survival rates differed significantly among the groups. Those results were validated in a Korean long-term study [19] and in a Western study [29], both suggesting that surgery is warranted in the high-risk group and that surgery should be avoided in multimorbid patients and low- and intermediate-risk groups according to eCura. Although these results are promising, there is a need for more prospective data for future guidance in decision making, especially in esophageal and colorectal cancers.

In recent years, the value of organ and function preservation has risen because of the functional burden and perioperative complications that come with major surgery. There are several case reports on laparoscopic lymph node dissection without gastrectomy in gastric cancer [30], showing uneventful postoperative courses in elderly patients. There are several trials on adjuvant radio chemotherapy after ESD when additional treatment is needed in early squamous cell esophageal carcinoma. The ECOG0508 trial showed 90.7% disease-specific survival in patients with T1a cancer with lymphovascular infiltration or T1b sm1–2 cancer over a period of three years [31]. Efficacy is comparable to surgery with a better safety profile and better organ preservation [32].

The role of salvage ESD after radio-chemotherapy in rectal lesions as well as in esophageal squamous cell lesions is discussed. In a recent study, the rate of successful salvage ESD in rectal cancer was 75% of cases after radio-chemotherapy, with 83% being initially staged as T3 [33]. Data on long-term outcomes are scarce in this scenario, but it seems to be a reasonable method for patients with contraindications to major surgery.

Despite new therapeutic concepts, we must conclude that the need for ESD in aging populations will rise and that ESD improves patients’ outcomes despite whether they meet the criteria for curability or not. As discussed above, there are many surgical studies showing that the rate of residual malignancy is low, and surgery does not improve long-term outcomes in elderly patients. Nishizawa et al. reported a five-year disease-specific survival of 93% after non-curative colorectal ESD without additional treatment in comparison to 94% with additional surgery [34].

A study on ESD-related complications in patients with comorbidities, defined as ASA ≥ 3, showed no significant difference between the ASA 3 and the ASA 1–2 groups [35]. Regarding long-term outcomes and mortality, there was a significant difference among the groups which, in our opinion, reflects the multimorbidity of elderly patients and the higher risk of non-disease-specific mortality.

## 5. Conclusions

There is a real need for guidelines for the individualization of patient management after non-curative ESD regarding the risk of lymph node metastasis, surgical complications, overall survival, and organ preservation. In our retrospective real-world analysis, no significant difference in cancer-associated mortality after ESD was observed despite a difference in malignant recurrence being observed among the groups. We therefore conclude that patients can benefit from ESD for early gastrointestinal malignancy, even if they do not meet the criteria for curative resection in comorbid patients, because of a reduction in cancer-related morbidity.

## Figures and Tables

**Table 1:** Overview of histopathological results of non-curative ESD, with multiple causes for non-curativeness possible. Abbreviations: m: intramucosal; Sm1–Sm3: submucosal infiltration depth; L: lymphatic invasion; V: vascular invasion; Bd: tumor budding; G1–3: tumor differentiation grading; R1: histologic residual malignancy in specimen margin.

	Esophagus	Stomach	Rectum
n	16	10	9
m	9	4	0
Sm1	2	2	3
Sm2	5	2	2
Sm3	0	2	4
L	2	4	4
V	0	1	2
Bd > 1	0	0	1
G1-2	8	6	8
G3	8	4	1
R1 horizontal	5	4	3
R1 vertical	3	2	2

**Table 2:** ESD histopathology of patient with malignant recurrence. Abbreviations: AC—adenocarcinoma; SCC—squamous cell carcinoma.

	Organ	Histopathology	Recurrence
1	Esophagus	AC; pT1a (m1) Rx basal L0 V0 Pn0 G2	Local
2	Esophagus	AC; pT1b (sm2) R1 basal L1 V0 Pn0 G3	Local
3	Stomach	pT1b (sm2) R0 L1 V0 Pn0 G3	Local
4	Esophagus	AC; pT1b (sm2) R1 basal L0 V0 Pn0 G2	Local
5	Esophagus	AC pT1a (m1) G1 Rx basal L0 V0 Pn0	Local
6	Esophagus	SCC pT1a (m3) L0 V0 Pn0 G3 R0	Lymph node
7	Stomach	pT1b (sm3) L0 V0 Pn0 Rx G1	local

**Table 3:** Outcomes of non-curative ESD (ncESD) and curative ESD (cESD). Abbreviations: FU—follow up; ASA—American Society of Anesthesiologists Score.

	ncESD	cESD	*p*
n	35	33	
Age (mean, SD)	77 (11.3)	72.9 (11.1)	0.12
Sex (% male)	80	73	0.337
ASA (mean)	2.4	2.2	0.56
Organ			
Esophagus	16	18	
Stomach	10	11	
Rectum	9	4	
FU period (months, mean, SD)	36.6 (27.1)	16.2 (12.1)	0.003
Tumor recurrence	7 (20%)	1 (3%)	0.017
Local	6	1	
Lymph node	1	0	
Deaths	3	1	0.33

## Data Availability

The data are not publicly available due to privacy restrictions.
